# Starch‐capped sulphur nanoparticles synthesised from bulk powder sulphur and their anti‐phytopathogenic activity against *Clavibacter sepedonicus*

**DOI:** 10.1049/nbt2.12044

**Published:** 2021-03-23

**Authors:** Marina Lesnichaya, Anastasiya Gazizova, Alla Perfileva, Olga Nozhkina, Irina Graskova, Boris Sukhov

**Affiliations:** ^1^ A. E. Favorsky Irkutsk Institute of Chemistry Siberian Branch Russian Academy of Sciences Irkutsk Russia; ^2^ Siberian Institute of Plant Physiology and Biochemistry Siberian Branch Russian Academy of Sciences Irkutsk Russia

## Abstract

Water‐soluble, stable nanoparticles of elemental sulphur with a size of 9‐52 nm have been synthesised using the stabilising potential of starch. Sulphide anions were used as sulphur precursors that were generated earlier from the bulk powder sulphur in the base‐reduction system NaOH‐N_2_H_4_·H_2_O followed by their oxidation with molecular oxygen to element sulphur atoms. Using a set of modern spectral and microscopic methods (XRD, optical spectroscopy, DLS, TEM), the phase state, elemental composition of the nanocomposites and their nanomorphological characteristics have been investigated. It was found that nanocomposites are formed as sulphur particles with the shape which is nearly spherical dispersed in the polysaccharide starch matrix with a pronounced tendency to cluster into ring formations. Water solubility and stability of the obtained nanoparticles is ensured by sorption of starch macromolecules on the surface of sulphur nanoparticles, with the thickness of the stabilising shell in a range of 10‐171 nm. In vitro experiments were carried out to study the anti‐microbial activity of the obtained sulphur nanocomposite (1.6% S) using the propidium iodide fluorescent dye staining method and the diffusion method. It showed that the water solution of the starch‐capped sulphur nanoparticles at the concentration of 6.25 µg/ml had a pronounced anti‐phytopathogenic activity against the potato ring rot pathogen *Clavibacter michiganensis* subsp. *sepedonicus*.

## 1 INTRODUCTION

Powder sulphur and its colloidal solutions have been actively used for several centuries as effective anti‐microbial [1], anti‐fungal [2], anti‐inflammatory [3] and disinfectant agents for the treatment of bacterial and fungal infections [4], auto‐immune diseases [5] and the treatment of agricultural plants [6]. In addition, sulphur is a biogenic element and is a component of sulphur‐containing amino acids, proteins, enzymes and coenzymes that forms the basis of the living matter [7, 8]. The biological activity of sulphur is determined by its ability to form disulphide bonds and sulfhydryl groups [9]. Disulphide bonds (‐S‐S‐) in polypeptide chains participate in creating the spatial structure of proteins. They bind amino acids to give a peptide and imparts it the unique structure, thus defining its basic physical and chemical properties. The cleavage of disulphide bonds leads to disorders of native protein structure and decreases its biological activity. Sulfhydryl groups or thiol compounds (‐SH) play an important role in functioning of active enzyme centres [10]. Having unique physical and chemical properties and the highest reactivity, the sulfhydryl groups easily undergo reversible red‐ox reaction under normal physiological conditions. Sulphur, as part of the sulfhydryl groups, is involved in the reactions of alkylation, acylation, thioldisulphide metabolism to deliver mercaptides (in reaction with heavy metal ions), mercaptals, mercaptols (in reaction with aldehydes and ketones), thereby contributing to the neutralisation of toxic substances in the body [11]. The sulfhydryl groups, which are components of coenzyme A and lipoic acid, participate in enzymatic reactions of formation and transfer of acyl residues associated with the lipid and carbohydrate metabolism. It has been proved that blocking of the sulfhydryl groups with specific reagents partially or completely inhibits the activity of many enzymes. The reactivity of sulphur is limited by its low bioavailability due to low water solubility. The use of sulphur in the ultra‐dispersed state significantly enhances its biological activity (anti‐microbial and anti‐fungal), decreasing the values of minimum inhibitory and minimum bactericidal concentrations, in particular, against *Staphylococcus aureus*, *Escherichia coli*, *Pseudomonas aeruginosa*, *Klebsiella pneumoniae*, *Acinetobacter baumannii*, *Stenotrophomonas maltophilia* and *Enterobacter aerogenes* microorganisms [12]. The improvement of anti‐microbial properties of sulphur with reduction of the particle size is probably due to a significant increase in their surface area [13]. At the same time, elemental sulphur nanoparticles (S^0^NPs) exert the most expressed biological properties owing to their extremely small size and the prevalence of surface reactive sulphur atoms over sulphur atoms in the volume of nanoparticles [14]. However, very high saturation energy of S^0^NPs surface determines their thermodynamic instability that leads to dense aggregation of nanoparticles and their precipitation from solution with the formation of massive sediments thus decreasing the chemical activity of S^0^NPs. One of the possible ways to provide the stability of the synthesized nanoparticles is their inclusion in the stabilising shell, which prevents aggregation of nanoparticles in the solution [15]. Various surfactants and polymers of synthetic or natural origin can be used as a stabilising shell [16]. In particular, *Chaudhuri RG* and *Paria S*, *Salem N*, et al., *Seitzhan T,* et al., *Kouzegaran VJ and Farhadi K* report on the successful application of Triton X‐100, sodium dodecyl sulphate, cetyl trimethylammoniumbromide, The aqueous extract of *Melia azedarach* leaves , sulfanol, sodium ligninsulphonate, sodium salt of polynaphthalene sulphonic acid, water soluble polymer sodium carboxymethylcellulose, saponin extracted from *A and Phylum Bracteatum* as effective stabilizers of S^0^NPs [17, 18, 19, 20, 21, 22]. However, the employment of plant extracts for the synthesis of S^0^NPs hinders standardisation and reproduction of the target nanomaterials due to heterogeneity of the chemical composition of the natural raw material, which depends strongly on the source and extraction method. Here, the use of naturally available polysaccharide, starch, as a stabilising matrix is proposed. Constant composition, cheapness, water solubility and stabilising potential determine the prospects of its application in the synthesis of biocompatible, water‐soluble nanomaterials [23]. So, the syntheses of the water‐soluble aggregate‐resistant nanoparticles of Ag, Au, Se and magnetite stabilised by starch macromolecules have been documented [24, 25, 26, 27, 28, 29, 30]. However, data on the synthesis of S^0^NPs in the starch matrix are currently missing. Inclusion of S^0^NPs in the starch shell will produce nanocomposites combining the biological properties of S^0^NPs (expressed anti‐microbial and fungicidal activity) with water solubility of starch. In addition, the method for the preparation of S^0^NPs from elemental sulphur powder, which is offered, can significantly reduce the cost of their production and get rid of a number of toxic reaction products. These water‐soluble, low‐toxic nanomaterials can be used in biology, medicine, agriculture, and in particular to fight the bacterial phytopathogen *Clavibacter michiganensis sepedonicus (Cms)* that causes potato ring rot [31]. Each year this phytopathogen destroys up to 60% of potato crop [32]. To date, there are no effective and safe means for humans and animals to fight *Cms*. The known anti‐microbial properties of S^0^NPs against *Cms* bacteria allow suggesting that the water‐solubility of S^0^NPs determined by the starch macromolecule shell will increase their bioavailability.

This article presents the results of the development of an effective method for the synthesis of a number of water‐soluble nanocomposites consisting nanoscale sulphur particles capped by polysaccharide starch matrix, followed by a detailed study of their structural and nanomorphological characteristics and testing of their bactericidal activity against bacterial phytopathogen *Cms*.

## 2 MATERIALS AND METHODS

### 2.1 Materials and reagents

In this work, water‐soluble starch from Sigma Aldrich, powder sulphur, sodium hydroxide and hydrazine hydrate (64%), ethyl alcohol (96%) were used. All reagents were purchased from Vekton (Russia) and were used as received without additional purification.

### 2.2 Synthesis of starch‐capped sulphur nanoparticles (general method)

Starch‐capped sulphur nanoparticles were synthesised by oxidation of sulphide anions generated from the elemental bulk sulphur in the base‐reduction system NaOH‐N_2_H_4_·H_2_O. So, in a three‐necked flask equipped with a reflux condenser and a thermometer, 1.0 g of NaOH and 2 ml of N_2_H_4_·H_2_O were placed upon constant stirring, after which the temperature of this reaction medium was brought to 70°C and 0.5 g of elemental bulk‐sulphur was added under an argon atmosphere. The synthesis time was 15 min. Next, an aliquot of the obtained reaction mixture containing S^2‐^‐anions (Vol. 200‐700 µL) was added to the previously obtained starch solution (1.0 g starch in 25 ml water) and stirred at heating (40°C) for 20 min. The isolation of nanocomposites and their purification from impurities was performed by precipitation with a four‐fold excess of EtOH, followed by repeated washing with ethanol and air drying at room temperature. Passivation of nanoparticles’ surface and their aggregative stability were achieved through its interaction with polar hydroxyl groups of starch. Elemental analysis: Starch ‐ C 42.42%, H 6.60%, O 50.98%; Starch/S^0^NPs (0.57% S) ‐ C 41.66%, H 5.74%, S 0.57%, O 52.03%; Starch/S^0^NPs (0.7% S) ‐ C 41.43%, H 6.29%, S 0.67%, O 51.61%; Starch/S^0^NPs (1.6% S) ‐ C 41.22%, H 6.60%, S 1.58%, O 50.60%. Yield of nanocomposites Starch/S^0^NPs: 89%‐98%%.

### 2.3 Characterisation of nanocomposite

#### 2.3.1 Transmission electron microscopy (TEM)

TEM measurements were performed on a Leo 906 E microscope operated at an accelerating voltage of 120 kV. The size distribution of nanoparticles was determined by statistical processing of TEM microphotographs.

#### 2.3.2 Optical spectroscopy of aqueous solutions of starch and nanocomposites Starch/S^0^NPs

The optical absorption spectra of aqueous solutions of starch and nanocomposites Starch/S^0^NPs were recorded on a Perkin Elmer Lambda 35 spectrophotometer in a quartz cuvette (path length, 1 cm).

#### 2.3.3 Elemental analysis (EA)

The elemental composition of starch and nanocomposites Starch/S^0^NPs was determined by a Thermo Scientific Flash 2000 CHNS analyser and by X‐ray energy dispersive microanalysis with a Hitachi TM 3000 scanning electron microscope equipped with a SDD XFlash 430‐4 X‐ray detector.

#### 2.3.4 X‐ray diffraction (XRD) analysis

X‐ray diffraction study was carried out on a Bruker D8 ADVANCE X‐ray diffractometer under monochromatised Cu‐Kα radiation mode Locked Coupled. The exposure time was 1 s for the phase analysis, and 3 s for the cell parameters and coherent lengths.

#### 2.3.5 Dynamic light scattering (DLS) and ζ‐potential

DLS measurements were carried out by using the Photocore Compact‐Z equipment at 25° ± 0.1°C. A software package DynaLS v2.0 (ALANGO) was used to calculate the correlation time distributions. Scattering angle was 90°, laser 654 nm, power 20 mV. The solutions were prepared at least 5 h before the measurements. In order to dispose dust, the scattering cells were washed with benzene, vacuumed, and filled with dust‐free air. The concentration of solutions was 0.4%. The polyelectrolyte velocity ν under external electric field E was measured. The electrophoretic mobility μE = ν/E was converted into the ζ‐potential (the potential of electrical double‐layer at the surface of hydrodynamic shear) by the Smoluchovsky equation μE = εε_o_ζ/ηs, where ε and ε_o_ are the dielectric permittivity of the solvent and vacuum, respectively. Each measurement was carried out 3 times, and the results were averaged.

### 2.4 Biological studies

The bactericidal activity of starch‐capped S^0^NPs was studied using *Cms* cultures. The Ac 1405 strain was derived from the All‐Russian collection of microorganisms of the G.K. Scriabin Institute of Biochemistry and Physiology of Microorganisms of the Russian Academy of Sciences. Bacteria were cultivated in a liquid medium consisting of peptone (10 g/L), yeast extract (5 g/L), glucose (5 g/L), sodium chloride (5 g/L). In the experiment, 10 ml of bacterial suspension grown during 3 days was used, to which 100 µL 0.1% aqueous solutions of starch or Starch/S^0^NPs nanocomposite (1.6% S) were applied pre‐filtered through a sterile syringe filter (Minisart NML) with a pore diameter 0.2 µm or the same volume of sterile water. The final concentration of sulphur in the bacterial suspension was 6.25 µg/ml. The bacteria were incubated together with the administered samples for 24 h, after which their viability was determined using a vitally fluorescent dye of propidium iodide at a final concentration of 7.5 µM. Cell viability was assessed by counting the dead cells coloured by propidium iodide in red and correlating the resulting number with the total number of cells in the field of view. At least 15 random fields of vision were used to count cells. The images were obtained using inverted fluorescent microscope Zeiss Axio Observer for microscopic analysis of bacterial cells after they were coloured by propidium iodide. The statistical processing of the obtained data was carried out using the STATISTICA 6.1 licence application package (Statsoft Inc., USA).

In addition, for the obtained nanocomposite Starch/S^0^NPs (1.6% S), the bactericidal activity against *Cms* was studied using the agar diffusion method. Sterile Petri dishes were placed on a strictly horizontal surface and a nutrient medium consisting of peptone (10 g/L), yeast extract (5 g/L), glucose (5 g/L), CaCO_3_ (5 g/L), microbiological agar (10 g/L) of 20 ml was poured into them to create an optimal layer thickness equal to 4 ‐ 5 mm. A thick layer of agar was sown with 50 µL of *Cms* bacterial suspension for 1 day and swabbed with a spatula until the microorganisms were evenly distributed over the entire surface of the Petri dish. Excess suspension was completely removed and dried for 30 min. Then holes (*d* = 6 mm) were drilled ("wells") at a distance of 2.5 cm from the centre of the Petri dish and at equal distances from each other, which were then filled with the objects under study (200 µL aqueous solution of starch, nutrient media or nanocomposite Starch/S^0^NPs (1.6% S)). Then, the Petri dishes were placed in the thermostat at 37°C without turning them over, strictly horizontally to form round areas. The results were evaluated after 5 days by the diameter of growth delay areas around the 'well', including the diameter of the 'well' itself. In some cases, oppressed areas were oval. In such cases, we were measured the largest and smallest zone diameters and calculated the average. The absence of delay of microbial growth around the 'well' indicates that this microorganism is not sensitive to testing drug. The experiment was carried out in 3‐fold reproducibility

## 3 RESULTS AND DISCUSSION

### 3.1 Synthesis of starch‐capped sulphur nanoparticles and characterisation of their structure and nanomorphological parameters

**FIGURE 1:**
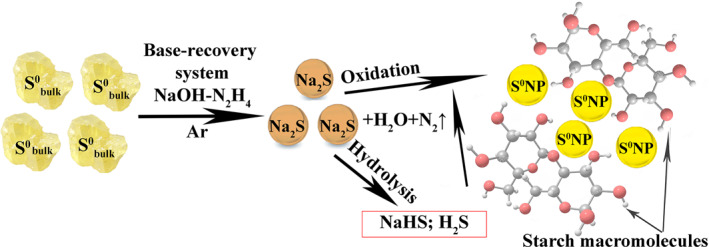
Supposed scheme of synthesis of starch‐capped sulphur nanoparticles from elemental sulphur powder

Synthesis of S^0^NPs was carried out in an aqueous starch solution via mild oxidation (without the use of specially added oxidizing agents) of sulphide ions pre‐generated by the reduction of powder elemental sulphur in the base‐reduction system ‘NaOH‐N_2_H_4_·H_2_O’ in an inert atmosphere. Sulphur atoms formed by the oxidation of sulphide‐anions coalesce with each other to afford S^0^NPs, which pass through all the main stages of nucleation, growth and maturation [33]. The starch macromolecules, in this case, stabilise the surface of the synthesised nanoparticles and provide for their aggregate stability. The main stages of sulphur nanocomposites formation are shown in Figure 1.

The sulphur content in the nanocomposites varied between 0.57% and 1.6% by changing the starch/S^2−^ ratio. It was found that the reduction reaction of elemental sulphur without alkali leads to formation of H_2_S. After introduction of S^2−^ions into the aqueous starch solution due to their low dissociation ability (pKa = 6.89), they are partially eliminated from reaction medium or oxidised with the formation of sulphur atoms that rapidly aggregate with each other without stabilisation, followed by sulphur sedimentation. This hinders the control of nanomorphological and structural characteristics of the resulting nanocomposites.

The use of alkali, in this case, is necessary for the directed production of sodium sulphide, which is easily hydrolysed in the starch aqueous solution to H_2_S and NaHS. Sodium hydrosulphide dissociates more easily (pKa = 12.91) than H_2_S and therefore dissolves in the reaction medium providing contact of the sulphide‐anions with dissolved oxygen acting as their oxidant. These features ensure favourable conditions for the gradual, controlled formation of elemental sulphur atoms, their coalescence in the nuclei of a new phase, growth and maturation and, finally, the generation of starch‐capped S^0^NPs.

**FIGURE 2:**
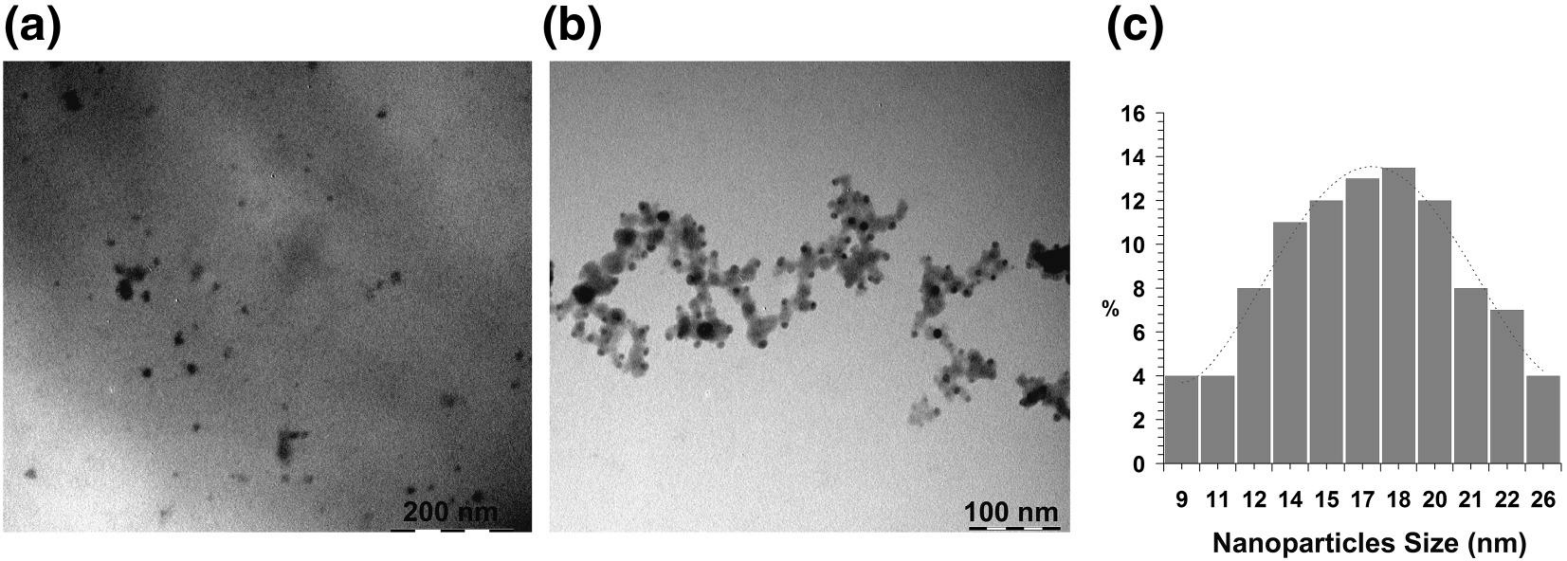
Microphotographs (a)*,* (b) and the diagram of disperse distribution of sulphur particles (c) in the starch‐based nanocomposite containing 0.57% S

According to the data of transmission electron microscopy, the nanocomposites are formed as sulphur nanoparticles dispersed in the starch matrix, whose shape is near to spherical (Figure 2‐a). Their average size and degree of polydispersion depend on the conditions of nanocomposite synthesis. The size of sulphur nanoparticles in the polysaccharide starch matrix varies between 9 and 52 nm, but some of nanoparticles represent no single particles well dispersed in the matrix, but are clustered into chains and rings (Figure 2‐b).

The stabilising potential of starch is determined by the presence of hydroxyl groups in its macromolecules. Presumably, starch hydroxyl groups interact (by means of the formation of intermolecular bonds) with uncompensated sulphur states having a charge on the surface of nanoparticles (due to a difference in energy potential of atoms in volume and on the surface). This provides surface passivation and prevents further aggregation of the nanoparticles. It has been found that the increase of "S^2−^/Starch" ratio is accompanied by increase of average size of the nanoparticles and their polydispersity. So, the increase of quantitative S^0^ content in the starch matrix from 0.57% to 1.58% augments the average size of the nanoparticles from 19 to 35 nm, respectively. Due to the fact that the concentration of the formed nuclei of new phase in the volume of the reaction medium is constant, the increase in concentration of sulphur precursor at constant concentration of a stabilizer promotes the gradual growth of the formed nuclei and maturation of nanoparticles with their increasing size. The enhancement of the ionic strength of the solution due to addition of Na^+^ ions into the reaction medium and the alteration of pH value change its rheological characteristics. Starch macromolecules reshape their conformation in the presence of higher amounts of sodium ions [34]. This increases viscosity of the reaction medium and diffusion limit of the transfer of the formed sulphur atoms from the oxidation area (S^2‐^‐2ē→S^0^) to the surface of growing particle. Appearance and upsurge of this kinetic barrier at a higher viscosity of the reaction medium increase the stochastic growth of the nanoparticles and, correspondingly, augment their polydispersion degree.

**FIGURE 3:**
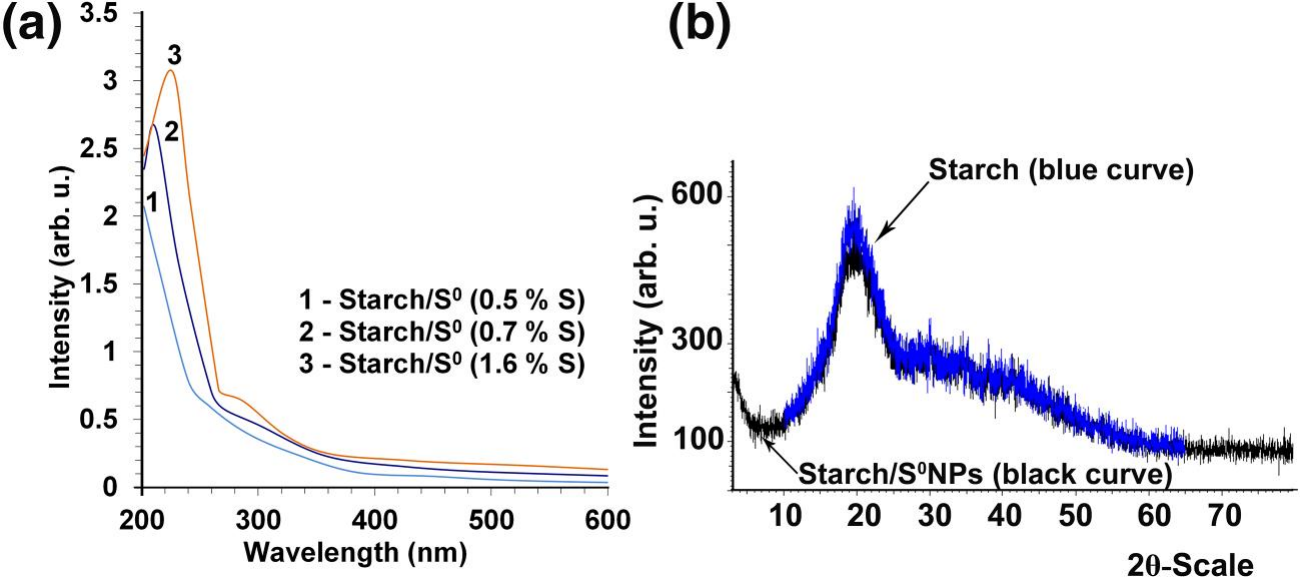
(a) ‐ Absorption spectra of 0.05% aqueous solutions of S^0^‐containing nanocomposites based on starch with different sulphur content; (b) ‐ diffractograms of initial starch and Starch/S^0^NPs nanocomposite (1.6% S)

Electronic absorption spectra of nanocomposites aqueous solutions in the UV‐ and visible spectral region are characterised by slow growth in the high energy region with a low‐intensity absorption maximum at 290 nm, typical for colloidal solutions of S^0^ [35]. The higher concentration of sulphur in the nanocomposites intensifies this maximum, thus confirming its assignment to electronic transitions on the surface of nanoparticles S^0^ (Figure 3‐a).

Using X‐ray diffraction analysis, it has been found that all obtained starch‐based nanocomposites are amorphous substances (Figure 3‐b). Their diffractograms almost completely repeat the diffractograms of the initial starch matrix. Multiple reflexes of elemental crystalline sulphur [36] are not present in the sample of the nanocomposite. Probably, sulphur reflexes do not appear on the background of wide reflexes from the starch matrix due to low sulphur content in the nanocomposite (up to 1.6%) and small size of the nanoparticles.

**FIGURE 4:**
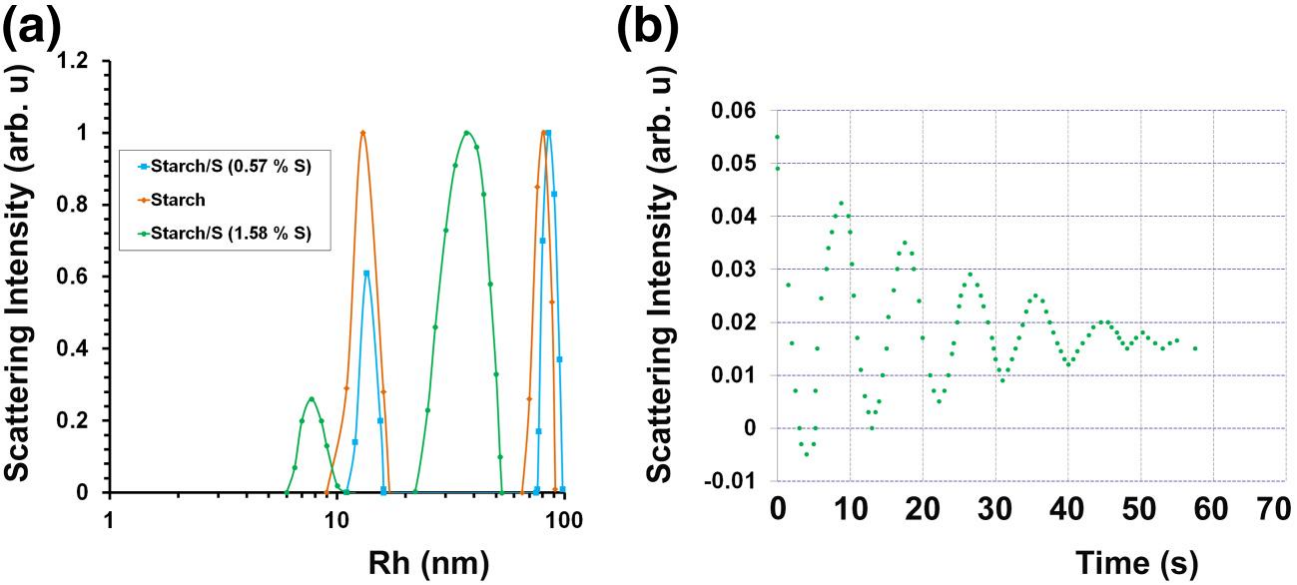
(a) ‐ Rh ‐distributions of 0.4% water solutions of the initial starch and nanocomposites Starch/S^0^NPs containing 0.57% and 1.58% sulphur; (b) ‐ autocorrelation function in electrophoretic light scattering of nanocomposite Starch/S^0^NPs (1.58% S^0^)

According to dynamic light scattering data, the starch aqueous solution contains two particle fractions with a hydrodynamic radius (Rh) of 13.5 and 85.0 nm in a ratio of 1:3 (Figure 4‐a).

Preliminary heating of the solution during sample preparation for DLS‐measurement probably promotes partial breakage of starch granules and leads to the release of amylose molecules. This is identified by the presence of a fast fraction of particles with Rh 13.5 nm in the solution, while the remaining (due to the short heating time) starch grains correspond to a slow fraction of particles with Rh 85.0 nm. Aqueous solutions of the nanocomposites are also characterised by the presence of two particle fractions, but the average size of Rh, as well as the ratio of these fractions significantly changes. Thus, addition of 0.57% S^0^NPs to the starch composition leads to a decrease in percentage of a fast fraction (0.219) and a small increase in Rh particles to 16.0 nm, whereas the content of slow particle fraction with Rh 90.0 nm increases to 0.780. The observed increase in the percentage of a slow fraction is probably due to the preservation of globular structure of starch granules owing to the absence of alkaline or thermal degradation of starch granules in the process of nanocomposite synthesis. At the same time, the introduction of 1.58% S^0^NPs into the starch composition decreases Rh values to 9.5 and 43.4 nm for the fast and slow fraction, respectively, and their ratio being 0.125:0.876. Presumably, such a decrease of Rh particles in the solution of nanocomposite is caused by alkaline degradation of starch in the process of sulphur nanocomposite synthesis due to the introduction of previously synthesised sulphide anions as Na_2_S into the reaction medium. Hydrolysis of sodium sulphide augments pH of the reaction medium and initiates the alkaline degradation of starch granules with amylose release and depolymerisation of its macromolecules [37]. Besides, one should bear in mind that in case of nanocomposites aqueous solutions, the particles fractions observed in this approach also belong to S^0^NPs, whose surface is covered by amylose or amylopectin macromolecules. Taking into account the size of S^0^NPs (9‐26 nm) (Figure 2‐c) determined by transmission electron microscopy, as well as their spherical shape, it can be assumed that the thickness of the stabilising starch shell differs between 2Rh (DLS) and the diameter of the nanoparticles (TEM) varies between 10.0 and 171.0 nm.

The stability of S^0^‐containing nanocomposites was evaluated by determining the ζ‐potential of their aqueous solutions. At the same time, ζ‐potential was established by the method of electrophoretic light scattering under the conditions of combination of two measurement modes: PALS (phase analysis light scattering) and ELS (electrophoretic light scattering). In the ELS mode, the autocorrelation function of the Doppler signal has a characteristic type of attenuation cosine (Figure 4b). Analysis of the distance between frequencies allows the calculation of the Doppler frequency and, consequently, the electrophoretic speed of particles. Meanwhile, the PALS mode allows to analyse the phase shift of the incident laser beam at light scattering due to particle movement. The particle velocity in the field calculated from the phase function permits to determinethe electrophoretic mobility of the particles Eq.(1).

(1) $$\mu_{E}=v/E$$

*v*‐velocities of charged particles in an electric field with voltage *E*.

Electrophoretical mobility of the particles is converted into ζ ‐potential using the Smolukhovsky's theory and application of corrections for different thicknesses of the double electric layer (Eq 2).

(2) $$\mu_{E}=2\epsilon \zeta /3\eta$$

ζ ‐potential.

μE‐electrophoretic mobility.

ε‐dielectric permittivity.

η‐viscosity.

It is found that ζ‐potential of the initial aqueous solution of starch and all nanocomposites obtained based on it varies from −9 mV (for the initial starch) to −20.8 mV (for the nanocomposite with 1.58% S^0^). The obtained values indicate that nanocomposites are sufficiently stable.

### 3.2 Study of the anti‐microbial properties of starch‐capped sulphur nanoparticles.

#### 3.2.1 In vitro testing of anti‐microbial activity of starch‐capped sulphur nanoparticles against Cms in the experiment with propidium iodide staining

**FIGURE 5:**
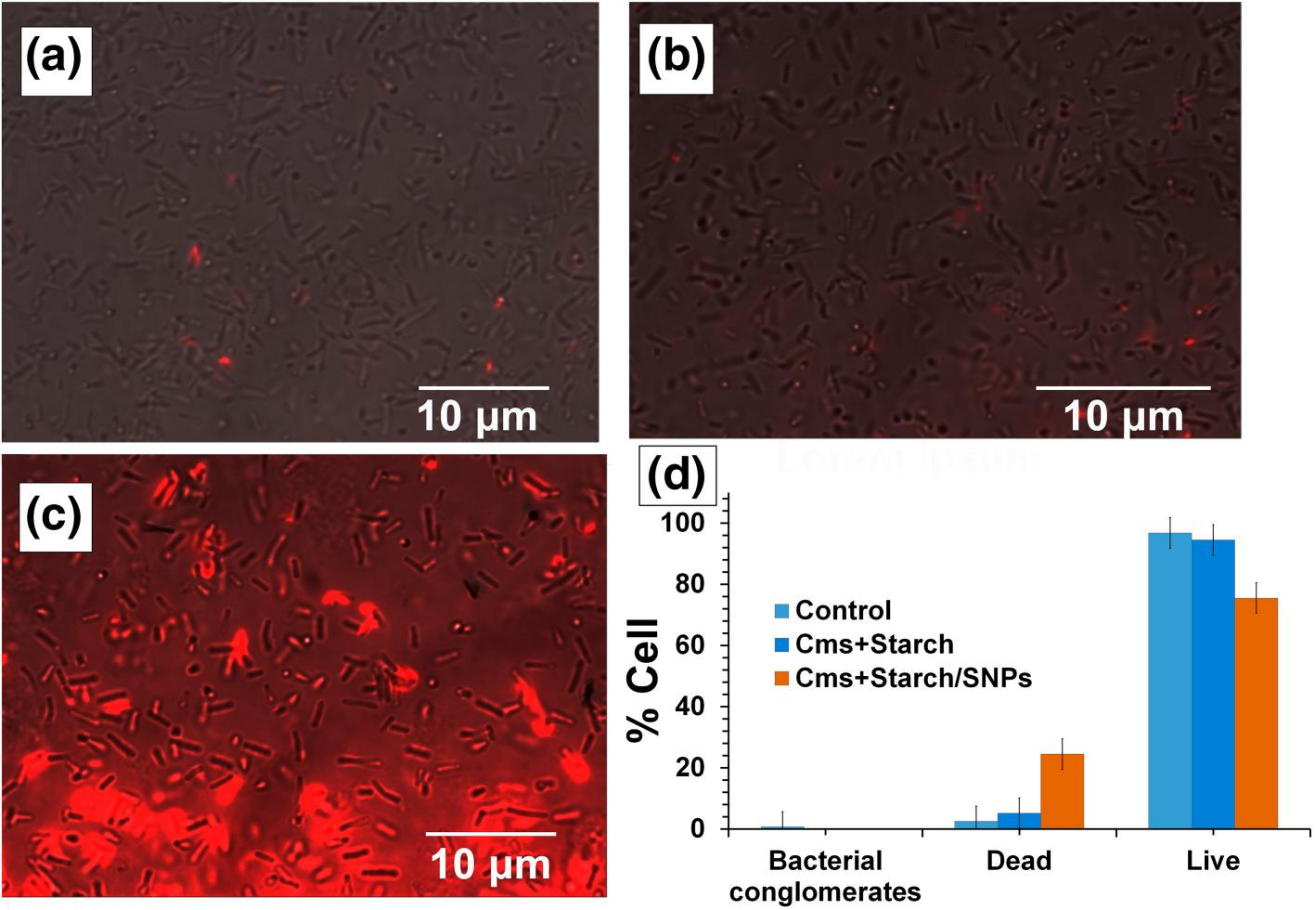
Fluorescence microscopic images of control sample of Cms culture ‐ (a) and Cms cultures after 24‐h exposition with solutions of starch ‐ (b) and Starch/S^0^NPs nanocomposite ‐ (c). (d) ‐ diagram of percentage distribution of conglomerate, live and dead forms of Cms cultures in conditions of their exposition with starch and nanocomposite Starch/S^0^NPs. The final concentrations of the active substances were 6.25 µg/ml (in terms of sulphur) and 0.018% for Starch/S^0^NPs and starch nanocomposite respectively

The viability of bacterial culture of *Cms* (Strain Ac 1405) on their interaction with starch‐stabilised S^0^NPs has been evaluated. The study was carried out by means of light and fluorescent microscopy for microscopic analysis of bacterial cells after colouring them with propidium iodide. At the same time, the fluorescent staining by this dye is typical only for dead cells of *Cms* due to the free passage of propidium iodide molecules through their damaged plasmatic membrane and its interaction with the nitrogen bases of intracellular nucleic acids [38]. Counting the coloured cells and attributing the obtained amount to a total number of *Cms* cells in the field of view allows estimating the degree of anti‐phytopathogenic action of the investigated nanocomposite. It is revealed that nanocomposite Starch/S^0^NPs has an inhibiting effect on the viability of *Cms* cells, causing their death, morphology changes, and partial incystation. Figure 5 presents images of propidium iodide staining initial *Cms* culture (a), and *Cms* cultivated together within 24 h with Starch/S^0^NPs nanocomposite (Figure 5‐c), as well as the initial starch (Figure 5‐b) for comparison. Red colouring of cells is typical for dead cells.

It is shown that the control sample of *Cms* cultures contains insignificant amounts of conglomerated and dead cells not exceeding 0.67% and 3.17%, respectively (Figure 5‐d). Morphologically, the culture represents a well‐differentiated low‐segmented rod cells. This indicates their viability and the possibility of using them to evaluate the phytopathogenic activity of the nanocomposite. The 24‐h incubation of *Cms* cultures with starch solution is accompanied by a slight (up to 5.18%) increase in *Cms* dead cells, while the exposition of *Cms* with a solution of Starch/S^0^NPs nanocomposite results in a massive death of *Cms* cells, up to 25% after their 24‐h co‐incubation. The image of *Cms* cultures obtained by means of fluorescence microscopy (Figure 5‐c) clearly shows a massive red glow from dead cells. The absence of significant differences in the number of dead *Cms* cells in the control group and the group incubated with starch confirms the absence of the anti‐phytopathogenic activity of starch, which is used as a stabilising shell for S^0^NPs. In the meantime, the detected anti‐microbial action of Starch/S^0^NPs nanocomposite indicates the determining role of S^0^NPs in their inhibitory action against *Cms*, even at low concentration. The detected anti‐phytopathogenic activity of Starch/S^0^NPs nanocomposite may be due to features of *Cms* metabolism, particularly the trophic attractiveness of starch. At the same time, S^0^NPs encapsulated in the starch shell cause the bactericidal effect of the nanocomposites by binding with protein and enzyme components of the cell and inactivating them.

#### 3.2.2 Study of the bactericidal effect of Starch/S^0^NPs nanocomposite on Clavibacter sepedonicus

**FIGURE 6:**
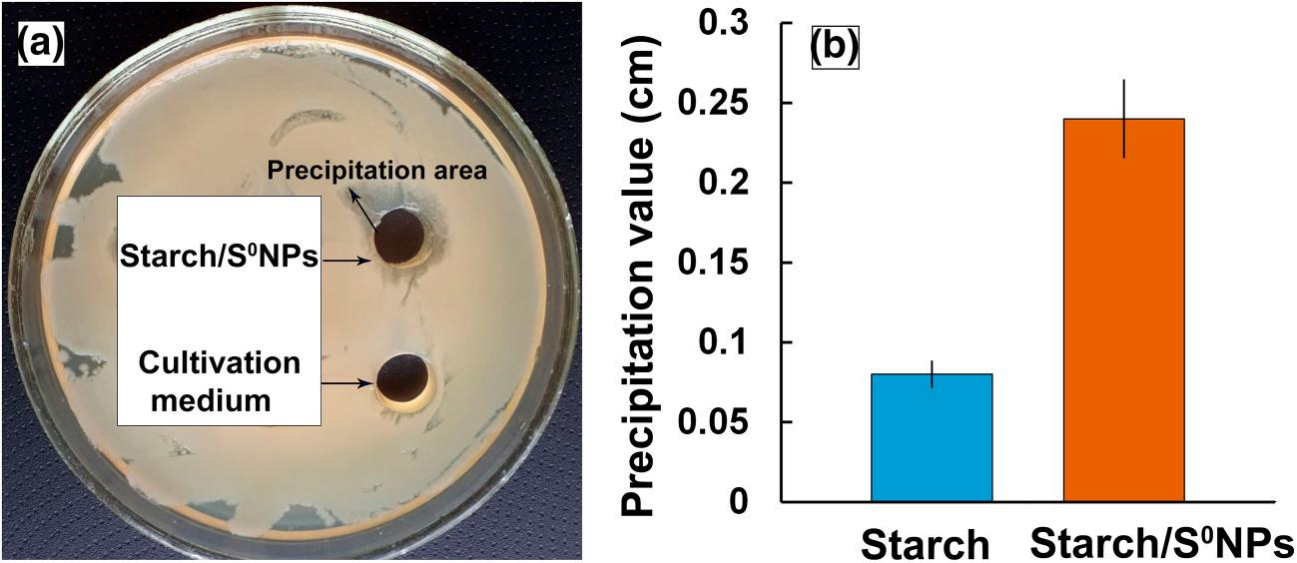
Image ‐ (a) and diagram ‐ (b), characterising the values of inhibition area of the Cms culture growth on the agar surface on their contact with the nutrient medium (for control) and aqueous solution of Starch/S^0^NPs nanocomposite (1.58% S) at a concentration of 6.25 µg/ml (in terms of sulphur)

The bactericidal activity of starch‐capped S^0^NPs has been studied by agar diffusion method (Figure 6‐a). The value of bactericidal activity correlates with the zone of pre‐precipitation and inhibition of bacterial growth in the area of contact with nanocomposite. It is found that in the well area containing the culture medium, no inhibition of bacterial growth occurs. This corresponds to the viability of *Cms* cells and determines the possibility of their use for testing the bactericidal activity of nanocomposite. Note that in the well area containing sterile aqueous solution of Starch/S^0^NPs nanocomposite at a concentration of 6.25 µg/ml, an active suppression of *Cms* growth takes place, value of precipitation zone being 0.22 cm (Figure 6‐b). The significantly lower precipitating area of the starch well also indicates the determining role of S^0^NPs in the bactericidal properties of the Starch/S^0^NPs nanocomposite. Probably, the major function of starch on the surface of S^0^NPs is, first of all, to ensure nanoscale size of sulphur and, consequently, to maintain water‐soluble and biocompatible properties. Secondly, starch macromolecules, being one of the main food sources for *Cms* bacteria, are likely to be captured by cells as a result of food capturing, delivered by S^0^NPs inside the cell.

The ability of sulphur (and especially sulphur in an ultra‐dispersed state) to interact with environmental components (in our case, with *Cms* products and nutrient medium) can be considered as a supposed mechanism of anti‐microbial activity of S^0^NPs. The result of this interaction may be a red‐ox reaction of available negatively charged S^2−^ ions on the nanoparticles’ surface with organic compounds in the cultural medium, with subsequent formation of either sulphides (in particular, endogenous H_2_S) or thiocarboxylic acids. Involvement of these sulphur‐containing compounds in cell metabolism of the *Cms* bacteria and their interaction with the surface of the bacterial cell can lead to inactivation of a number of enzymes, components of cell membranes, damage to DNA, increased oxidative stress and finally a programed cell death [39, 40].

## 4 CONCLUSION

Thus, an available, environmentally friendly method has been developed for the synthesis of water‐soluble aggregate‐stable sulphur nanoparticles capped by starch. The resulting starch‐capped sulphur nanoparticles are characterised by high stability (ζ‐potential 20.8 mV) and expressed anti‐phytopathogenic action against *Cms* in the working concentration of 6.25 µg/ml. The starch shell on the surface of the nanoparticles determines the water solubility and stability of the resulting sulphur nanoparticles, and is likely to contribute to the effective food capture of sulphur nanoparticles by bacterial cells. The obtained data can be used for further development (on the basis of synthesised nanoparticles) of tools to control the potato ring rot and other agricultural plant diseases caused by the *Cms* bacteria.
